# Sulforaphane Protects against High Cholesterol-Induced Mitochondrial Bioenergetics Impairments, Inflammation, and Oxidative Stress and Preserves Pancreatic *β*-Cells Function

**DOI:** 10.1155/2017/3839756

**Published:** 2017-03-12

**Authors:** Catalina Carrasco-Pozo, Kah Ni Tan, Martin Gotteland, Karin Borges

**Affiliations:** ^1^Department of Nutrition, Faculty of Medicine, University of Chile, P.O. Box 8380453, Santiago, Chile; ^2^School of Biomedical Sciences, The University of Queensland, Brisbane, QLD 4072, Australia; ^3^Institute of Nutrition and Food Technology, University of Chile, P.O. Box 138-11, Santiago, Chile

## Abstract

Cholesterol plays an important role in inducing pancreatic *β*-cell dysfunction, leading to an impaired insulin secretory response to glucose. This study aimed to determine the protective effects of sulforaphane, a natural isothiocyanate Nrf2-inducer, against cholesterol-induced pancreatic *β*-cells dysfunction, through molecular and cellular mechanisms involving mitochondrial bioenergetics. Sulforaphane prevented cholesterol-induced alterations in the coupling efficiency of mitochondrial respiration, improving ATP turnover and spare capacity, and averted the impairment of the electron flow at complexes I, II, and IV. Sulforaphane also attenuated the cholesterol-induced activation of the NF*κ*B pathway, normalizing the expression of pro- and anti-inflammatory cytokines. In addition, it also inhibited the decrease in* sirtuin 1* expression and greatly increased* Pgc-1α* expression in Min6 cells. Sulforaphane increased the expression of antioxidant enzymes downstream of the Nrf2 pathway and prevented lipid peroxidation induced by cholesterol. The antioxidant and anti-inflammatory properties of sulforaphane and its ability to protect and improve mitochondrial bioenergetic function contribute to its protective action against cholesterol-induced pancreatic *β*-cell dysfunction. Our data provide a scientifically tested foundation upon which sulforaphane can be developed as nutraceutical to preserve *β*-cell function and eventually control hyperglycemia.

## 1. Introduction

Sulforaphane (SFN) is a natural isothiocyanate derived from a glucosinolate found in cruciferous vegetables, especially broccoli, and is a potent activator of several NF-E2-related factor-2- (Nrf2-) regulated phase 2 enzymes [1]. Treatment of pancreatic *β*-cell lines, RINm5F, and INS-1(832/13) with SFN increases Nrf2 nuclear translocation and expression of phase 2 enzyme genes such as heme oxygenase-1* (Hmox-1)* and glutamate-cysteine ligase catalytic subunit* (Gclc)* [2, 3]. Activation of Nrf2 by SFN prevented *β*-cell damage induced by cytokines downstream of the NF*κ*B signaling pathway and restored insulin secreting responses to glucose [2]. Furthermore, pretreatment with SFN blocked the development of type 1 diabetes in streptozotocin-treated animals [2, 4]. It has also been shown that the activation of Nrf2 protects mitochondria from dysfunction and promotes mitochondrial biogenesis in neurodegenerative diseases [5]. In fact, recently we have shown that SFN is anticonvulsant and improves mitochondrial function in mouse hippocampal formations [6]. However, the effect of SFN on the mitochondrial function related to pancreatic *β*-cell function has not been studied yet.

Cholesterol is an essential structural component of cell membranes and serves as a precursor for the biosynthesis of steroid hormones and bile acids. High levels of cholesterol, however, contribute to pancreatic *β*-cell dysfunction [7, 8], leading to an impaired insulin secretory response to glucose, which is a hallmark of the transition from prediabetic to diabetic state [9]. Mice with specific inactivation of ABCA1 (ATP-binding cassette transporter subfamily A member 1), a transporter that mediates reverse cholesterol efflux in *β*-cells [10], and mice with LXR-*β* (liver X receptor beta) deficiency [11], a nuclear hormone receptor that increases ABCA1 expression in response to cholesterol, show impaired glucose tolerance and insulin secretion. Moreover, a direct link has been found between elevated cholesterol and reduced insulin secretion in islets isolated from C57BL/6J mice, in INS-1 rat pancreatic *β*-cells [12], and in Min6 cells [7, 8], whereby insulin secretion can be restored through cholesterol depletion [12]. Elevated cholesterol levels in pancreatic islets are also associated with *β*-cell dysfunction and reduced glucose-stimulated insulin secretion (GSIS) in LDL receptor deficient mice [13]. Given that pancreatic *β*-cells are particularly susceptible to oxidative stress due to their low antioxidant defenses [14], it has been suggested that cholesterol may induce *β*-cell dysfunction by increasing oxidative stress and causing mitochondrial damage [7, 8, 15, 16]. Although cholesterol has been shown to increase TNF-*α* (tumor necrosis factor alpha), interleukin-6 (IL-6), and macrophage colony-stimulating factor (M-CSF) in macrophages [17], it remains to be established to which extent inflammation contributes to *β*-cells dysfunction induced by cholesterol.

The present study aimed to determine the mechanism underlying the protective effect of SFN on impaired GSIS in a pancreatic *β*-cell line exposed to high concentration of cholesterol. This study also addressed the protective effects of SFN on mitochondrial bioenergetic dysfunction, inflammation, and oxidative stress induced by high levels of cholesterol and the molecular pathways involved. The effects of SFN were compared to those induced by 3,4-dihydroxyphenylacetic acid (ES), a microbiota-derived metabolite of quercetin that induces Nrf2 activation and that has been shown to protect against pancreatic *β*-cells dysfunction induced by high cholesterol [7].

## 2. Materials and Methods

### 2.1. Materials

3,4-dihydroxyphenylacetic acid (ES), water-soluble cholesterol, carbonyl cyanide-4-(trifluoromethoxy)phenylhydrazone (FCCP), glucose, L-glutamine, pyruvate, sucrose, mannitol, fatty acid-free BSA, oligomycin, antimycin A, rotenone, N,N,N′,N′-tetramethyl-p-phenylenediamine (TMPD), ascorbic acid (AA), succinate, and malate were purchased from Sigma (MO, USA). Sulforaphane (SFN) was from Sapphire Bioscience Pty Ltd., (Sydney, NSW, Australia). Cytokine/Chemokine Magnetic Bead Panel MCYTOMAG-70K was from EMD Millipore Corporation (MI, USA). The TBARS Kit Assay, NF*κ*B (p65) Transcription Factor Assay, and Nuclear Extraction Kit were obtained from Cayman Chemicals (MI, USA). The Rat Insulin ELISA Kit was from ALPCO (MA, USA). RNeasy Mini Kit was from Qiagen (NRW, Germany). RQ1 RNase-free DNase Kit was from Promega (WI, USA). Tetro cDNA Synthesis Kit was from Bioline (NSW, Australia). SYBR Green PCR Master Mix was from Applied Biosystems (CA, USA). The M-MLV Reverse Transcriptase, Oligo(dT)15 Primer, and Recombinant RNasin Ribonuclease Inhibitor were from Promega (WI, USA). The Pierce BCA Protein Assay Kit was from Thermo Scientific (IL, USA). All cell culture reagents (DMEM, FBS, penicillin, and streptomycin) were from GIBCO BRL (NY, USA).

### 2.2. Cell Culture

Min6 cells (p38-p51) (kindly provided by Dr. Francisco Pérez, University of Chile) were cultured in DMEM (25 mM glucose) supplemented with 10% heat-inactivated FBS, 100 IU/ml penicillin, 100 *µ*g/ml streptomycin in a humidified atmosphere of 95% air, and 5% CO_2_. All the experiments were conducted in nonsupplemented DMEM medium. Protein content was determined by using the Pierce BCA Protein Assay Kit. The absorbance and fluorescence were measured using a Multi-Mode Microplate Reader (Synergy HT, BioTek, VT, USA). The “water-soluble cholesterol” containing 47 mg of cholesterol/g solid according to Certificate of Analysis (molar ratio, 1 : 6 cholesterol/methyl-*β*-cyclodextrin) was used to deliver cholesterol to the cells, as previously described [7, 8, 15, 18, 19]. Considering that methyl-*β*-cyclodextrin induces cholesterol depletion from cell membranes at very high concentration (~2% or 5 mM) [18, 20], a 10-time lower concentration of this compound was used here. Similar to previous studies we incubated Min6 cells for 6 h with 320 *µ*M of cholesterol [7, 8, 15].

### 2.3. Mitochondrial Coupling Assay

Using the extracellular flux XF^e^96 analyzer (Seahorse Bioscience, MA, USA), the degree of coupling between the electron transport chain (ETC), the oxidative phosphorylation machinery, and ATP production was evaluated. Min6 cells plated at 1 × 10^5^ cells/well on XF^e^96 well plates were treated for 6 h with cholesterol with/without SFN or ES, and oxygen consumption rate (OCR) was measured. After washing, the cells were incubated for 1 h at 37°C in XF Assay Modified Medium containing 25 mM glucose, 2 mM L-glutamine, and 2 mM pyruvate, pH 7.4. The same study was conducted in the absence of glucose, with XF Assay Modified Medium containing 2 mM L-glutamine and 2 mM pyruvate, pH 7.4. State 3 (basal respiration), state 4o (induced with 2 *μ*M oligomycin), and maximal respiration (state 3u, stimulated with 1.5 *μ*M FCCP, an uncoupling agent) were sequentially measured. Maximal respiration, spare capacity (state 3u minus state 3), ATP turnover (state 3 minus state 4o), and coupling efficiency (ATP turnover/state 3) were calculated as previously described [8]. All values were normalized to protein content. Rotenone and antimycin A (1 *μ*M each) were added to block complex I and III, respectively, in order to determine the OCR unrelated to mitochondrial oxygen consumption. Only the mitochondrial dependent OCRs were considered for the calculation of mitochondrial function parameters which are expressed as nmol of oxygen consumed/min/mg protein.

### 2.4. Mitochondrial Electron Flow

The sequential electron flow through the complexes of the ETC was studied in mitochondria isolated from Min6 cells treated with cholesterol in the absence or in the presence of SFN or ES for 6 h, by using the extracellular flux XF^e^96 analyzer. This assay allows the study of the contribution and function of complexes I, II, and IV in the ETC in terms of OCR. Mitochondria were isolated as previously described [6, 7, 21]. Briefly, cells were harvested, washed in a Ca^2+^/Mg^2+^-free PBS, and centrifuged (10 min; 1,000*g*; 4°C). The pellet was resuspended and homogenized in MSHE solution (70 mM sucrose, 210 mM mannitol and 5 mM HEPES, 1 mM EGTA, and 0.5% (w/v) fatty acid-free BSA, pH 7.2). The homogenate was centrifuged at 1,000*g* for 10 min at 4°C and the resulting supernatant at 12,000*g* for 10 min. The pellet was washed and centrifuged at 10,000*g* for 10 min and resuspended in the same buffer. This preparation was used immediately for electron flow assay.

Freshly isolated mitochondria (4 *µ*g) were plated in each well of XFe96 well plate and centrifuged at 2,000*g* for 20 min at 4°C in MAS buffer (70 mM sucrose, 220 mM mannitol, 10 mM KH_2_PO_4_, 5 mM MgCl_2_, 2 mM HEPES, 1 mM EGTA, and 0.2% (w/v) fatty acid-free BSA, pH 7.2) in the presence of 10 mM pyruvate, 2 mM malate, and 4 *µ*M FCCP (state 3u). Mitochondria were activated by the addition of 6 volumes of MAS at 37°C. Subsequently 2 *µ*M rotenone (an inhibitor of complex I), 10 mM succinate (a substrate for complex II), 4 *µ*M antimycin A (an inhibitor of complex III), and 1 mM AA/100 *µ*M TMPD (AA/TMPD act as electron donors to cytochrome C in complex IV) were sequentially added. Rotenone inhibited the oxidation of pyruvate/malate mediated via complex I and, thus, the contribution of complex I to respiration was calculated as state 3u minus OCR after rotenone injection. Injection of succinate allows the mitochondria to respire via complex II; thus complex II-driven respiration was calculated as the increase in OCR after succinate injection. Electron flow is inhibited at complex III by antimycin A; then after the addition of AA/TMPD the activity of complex IV was calculated [22]. Values from electron flow assay are expressed as nmol of oxygen consumed/min/mg protein.

### 2.5. qPCR Measurements

Gene expression related to mitochondrial function and downstream of Nrf2 activation was studied. The mRNA levels of* Sirt1, *peroxisome proliferator-activated receptor gamma coactivator-1-alpha* (Pgc-1α), Hmox-1, Gclc *(the limiting enzyme in glutathione synthesis), and Cu/Zn superoxide dismutase* (Sod1)* were evaluated using qPCR as previously described [8, 23] in Min6 cells treated for 20 h with cholesterol in the presence or in absence of SFN or ES. The relative fold expression of each gene is expressed relative to the cycle thresholds of two housekeeping genes,* Hmbs* and* Tbp *in Min6 cells. Primer sequences are provided in Table 2.

### 2.6. Lipid Peroxidation

Oxidative stress in Min6 cells was evaluated by measuring lipid peroxidation using a TBARS Assay Kit according to manufacturer's instructions. Cells were plated in 75 cm^2^ flasks at a density of 2 × 10^7^ cells 24 h prior to incubation with cholesterol in the presence or absence of SFN or ES for 6 h.

### 2.7. Inflammatory Status Evaluation

Pro- and anti-inflammatory cytokines were measured in cell homogenates from Min6 treated for 20 h with cholesterol in the presence or absence of SFN or ES by using the MCYTOMAG-70K Cytokine/Chemokine Magnetic Bead Panel. We also evaluated the NF*κ*B activation in nuclear protein extracts from Min6 cells treated with cholesterol and/or SFN or ES for 6 h. Nuclear extracts were obtained using a Nuclear Extraction Kit (Cayman). An NF*κ*B (p65) Transcription Factor Assay Kit (Cayman) was used to evaluate NF*κ*B p50 and p65 DNA binding activities to the response element by ELISA. The values were normalized to protein content.

### 2.8. Insulin Secretion Assay

Insulin secretion assay was performed as we previously described [7, 8]. Briefly, Min6 cells (p38–41) were incubated with cholesterol in the presence or absence of SFN or ES for 6 h, in serum-free and glucose-free DMEM supplemented with 2 mM L-glutamine and 25 mM HEPES, pH 7.4. Cells were then washed twice with PBS and placed immediately in serum-free DMEM with low glucose (5 mM) or high glucose (25 mM) for 1 h and medium was collected for the GSIS assay, according to Suzuki et al. [24]. Insulin levels in the medium were measured using a Rat Ultrasensitive Insulin ELISA Kit according to the manufacturer's instructions. Basal insulin secretion was measured in the absence of glucose in Min6 cells exposed to the different treatments. All values were normalized to protein content.

### 2.9. Statistical Analysis

Data were analyzed by two-way ANOVA (specified in results and each figure legend), followed by Bonferroni's Multiple Comparison Test using GraphPad Prism 6 statistical software (La Jolla, CA, USA). Unless indicated otherwise, the experiments were performed three times (three independent culture preparations) and in triplicate or quadruplicate. Values with different superscript letters (A, B, C, D, E, and F) indicate significant differences (*p* < 0.05) between groups. Values are expressed as mean ± SEM.

## 3. Results

### 3.1. Sulforaphane Prevents Cholesterol-Induced Mitochondrial Bioenergetic Dysfunction

**Figure 1:**
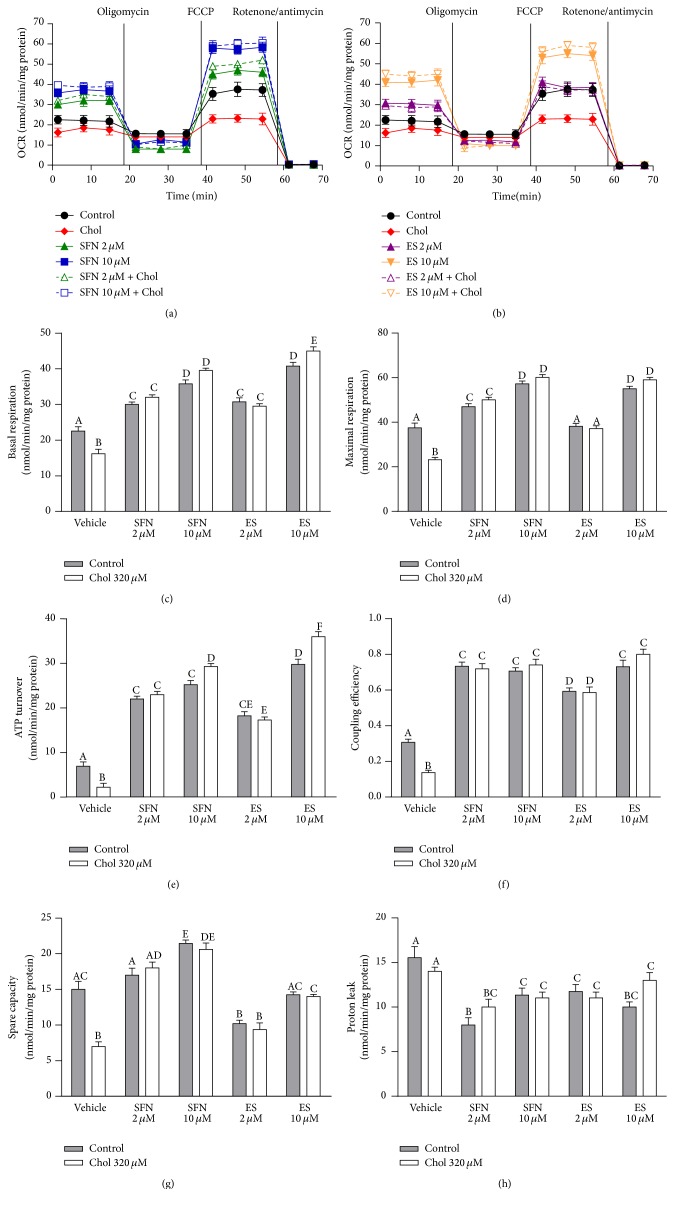
SFN improves mitochondrial bioenergetics in Min6 cells exposed to cholesterol. Min6 cells were incubated with 320 *µ*M of cholesterol and/or 2 or 10 *µ*M of SFN or ES. Mitochondrial functions with SFN (a) or ES (b) treatment were measured in the form of OCR and expressed as nmol of oxygen consumed/min/mg of protein using the XF^e^96 analyzer in cells treated for 6 h with vehicle (black solid circle) or with 320 *µ*M cholesterol (chol) (red solid diamond), 2 *µ*M SFN (green solid triangle), 10 *µ*M SFN (blue solid square), 2 *µ*M SFN + 320 *µ*M chol (green vacant triangle), 10 *µ*M SFN + 320 *µ*M chol (blue vacant square), 2 *µ*M ES (violet solid triangle), 10 *µ*M ES (orange solid triangle), 2 *µ*M ES + 320 *µ*M chol (violet vacant triangle), and 10 *µ*M ES + 320 *µ*M chol (orange vacant triangle). (c) Basal respiration and (d) maximal respiration stimulated with 1.5 *μ*M FCCP (State 3u) are shown. (e) ATP turnover was calculated as basal respiration minus respiration induced with 2 *μ*M oligomycin (State 4o). (f) Coupling efficiency was calculated as the ratio of basal respiration minus State 4o relative to basal respiration. (g) Spare capacity was calculated as maximal respiration minus basal respiration. (h) Proton leak was calculated as State 4o minus nonmitochondrial OCR induced with 1 *μ*M antimycin/rotenone. Control cells were not treated with cholesterol. All values are expressed as mean ± SEM, from three independent culture preparations, each treatment performed in quadruplicate. All two-ways ANOVAs and Bonferroni posttest. Values with different superscript letters indicate significant differences (*p* < 0.05) between groups. Chol: cholesterol; SFN: sulforaphane; ES: 3,4-dihydroxyphenylacetic acid; and OCR: oxygen consumption rate.

Results of the coupling assay measuring OCRs in Min6 cells with the XF^e^96 analyzer are shown in Figure 1(a) for SFN and in Figure 1(b) for ES. Mitochondrial function parameters were quantified in Figures 1(c)–1(h). Cholesterol treatment in Min6 cells decreased basal respiration by 30% (Figure 1(c)), maximal respiration by 38% (Figure 1(d)), ATP turnover by 68% (Figure 1(e)), coupling efficiency by 55% (Figure 1(f)), and spare capacity by 53% (Figure 1(g)), but it did not alter proton leak (Figure 1(h)) compared to cells which were not treated with cholesterol (all two-way ANOVAs, post-tests *p* < 0.05, Figures 1(c)–1(g)). SFN and ES prevented the mitochondrial OCR impairments induced by cholesterol and improved the mitochondrial functions in both cholesterol treated cells and control cells in a concentration-dependent manner. SFN at both 2 *µ*M and 10 *µ*M, in the absence or presence of cholesterol, also improved the basal respiration by 33% and 67% (Figure 1(c)) and the maximal respiration by 28% and 56% (Figure 1(d)), respectively. SFN at both 2 and 10 *µ*M increased ATP turnover and coupling efficiency by 210% and 130%, respectively, in comparison with untreated cells (Figures 1(e) and 1(f)). SFN 10 *µ*M also enhanced the spare capacity by 45% (Figure 1(g)). In the presence or absence of cholesterol, ES at 2 *µ*M and 10 *µ*M expanded basal respiration by 35% and 90%, ATP turnover by 163% and 380% and coupling efficiency by 93% and 150%, respectively in comparison with untreated cells (Figures 1(c), 1(e), and 1(f)). ES at 10 *µ*M, with or without cholesterol, improved the maximal respiration by 50% (Figure 1(d)) and both SFN and ES decreased the proton leak by around 30% with respect to the control cells (Figure 1(h)). No significant differences were found in the mitochondrial function parameters between the treatments when this coupling assay was conducted in the absence of glucose (data not shown).

**Figure 2:**
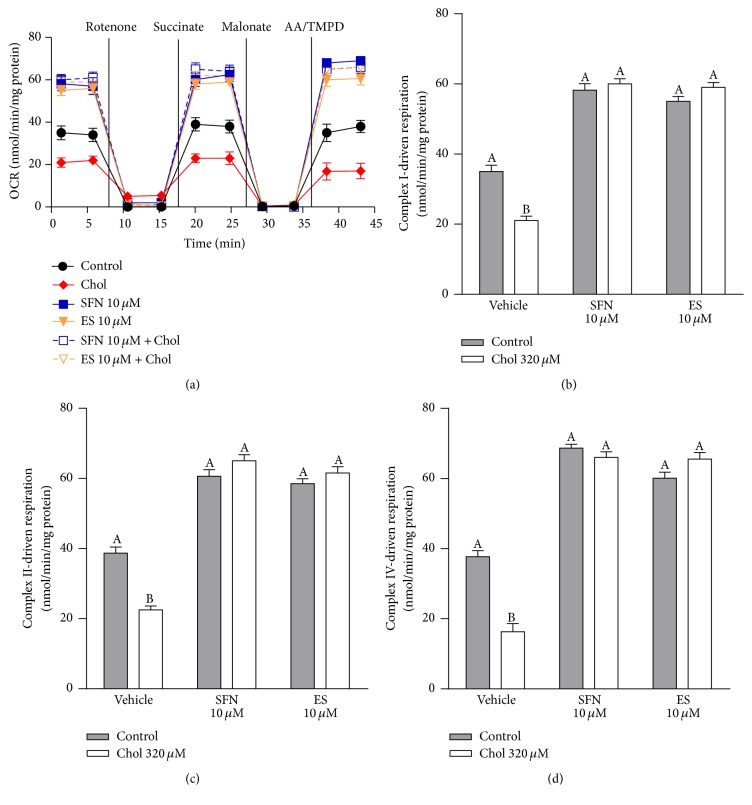
SFN increases mitochondrial electron flow in Min6 cells exposed to cholesterol. Min6 cells were incubated with 320 *µ*M of cholesterol and/or 10 *µ*M of SFN or ES and mitochondria were isolated. (a) Electron flow was measured in the form of OCR and expressed as nmol of oxygen consumed/min/mg of protein using the XF^e^96 analyzer in mitochondria from control cells (black solid circle) or cells after 6 h treatment with: 320 *µ*M cholesterol (chol) (red solid diamond), 10 *µ*M SFN (blue solid square), 10 *µ*M SFN + 320 *µ*M chol (blue vacant square), 10 *µ*M ES (orange solid triangle), and 10 *µ*M ES + 320 *µ*M chol (orange vacant triangle). (b) Respiration driven by complex I was calculated as from the decrease in OCR after the addition of 2 *μ*M rotenone. (c) Complex II- and (d) IV-driven respiration was calculated as the increase in OCR after the addition of 10 mM succinate or 1 mM AA/10 *μ*M TMPD, respectively. Control cells were not treated with cholesterol. Values are expressed as mean ± SEM, from three independent culture preparations, each treatment performed in quadruplicate. All two-way ANOVAs and Bonferroni posttests. Values with different superscript letters indicate significant differences (*p* < 0.05) between groups. AA: ascorbate; Chol: cholesterol; SFN: sulforaphane; ES: 3,4-dihydroxyphenylacetic acid; and OCR: oxygen consumption rate.

The electron flow assay measured as OCR in isolated mitochondria from Min6 is shown in Figure 2(a). Cholesterol pretreatment decreased the respiration driven by complex I, complex II, and complex IV by 51% (Figure 2(b)), 42% (Figure 2(c)), and 57% (Figure 2(d), all two-way ANOVAs, post-tests *p* < 0.05, Figures 2(b)–2(d)). Both SFN and ES at 10 *µ*M, in the presence or absence of cholesterol, improved respiration due to the increased activity of complex I by 66% and 62% (Figure 2(b)), complex II by 62% and 59% (Figure 2(c)), and complex IV by 79% and 67% (Figure 2(d)), respectively.

### 3.2. Sulforaphane and Cholesterol Modulate Gene Expression Related to Mitochondrial Function

**Figure 3:**
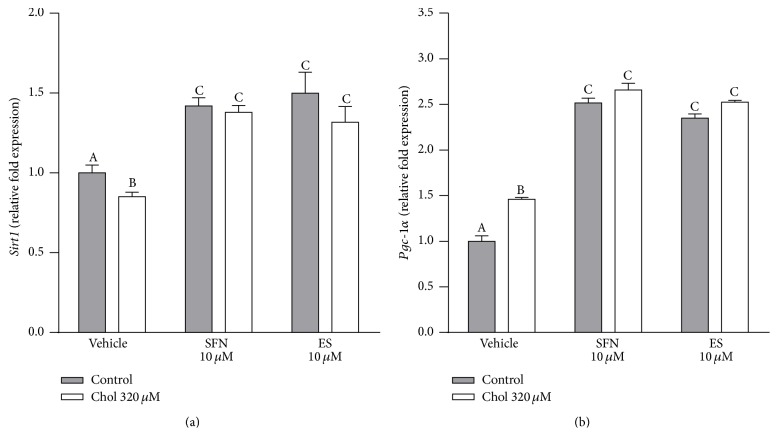
SFN induces the expression of genes regulating mitochondrial function. Gene expression of (a)* Sirt1* and (b)* Pgc-1α* in Min6 cells treated with 320 *µ*M of cholesterol and/or 10 *µ*M of SFN or ES for 20 h was evaluated. Values are expressed as relative fold expression of two housekeeping genes (Table 2). Values are expressed as mean ± SEM, from three independent culture preparations, each treatment performed in quadruplicate. All two-ways ANOVAs and Bonferroni post-tests. Values with different superscript letters indicate significant differences (*p* < 0.05) between groups. Chol: cholesterol; SFN: sulforaphane; ES: 3,4-dihydroxyphenylacetic acid; Sirt1: sirtuin 1; and Pgc-1*α*: peroxisome proliferator-activated receptor gamma coactivator-1-alpha.

Sirtuin 1 and PCG-1*α* are key regulators of mitochondrial function [25]. Cholesterol treatment decreased* Sirt1* gene expression by 15%, while SFN or ES, in the presence or absence of cholesterol, increased its expression by around 40% compared to vehicle treated Min6 cells (two-way ANOVA, posttest *p* < 0.05, Figure 3(a)). Cholesterol increased* Pgc-1α* gene expression by 46%, while SFN or ES at 10 *µ*M, in the presence or absence of cholesterol, increased the expression by around 140% (two-way ANOVA, posttest *p* < 0.05, Figure 3(b)).

### 3.3. Sulforaphane Prevents Cholesterol-Induced Lipid Peroxidation and Induces the Expression of Genes Related to Antioxidant Defences

**Figure 4:**
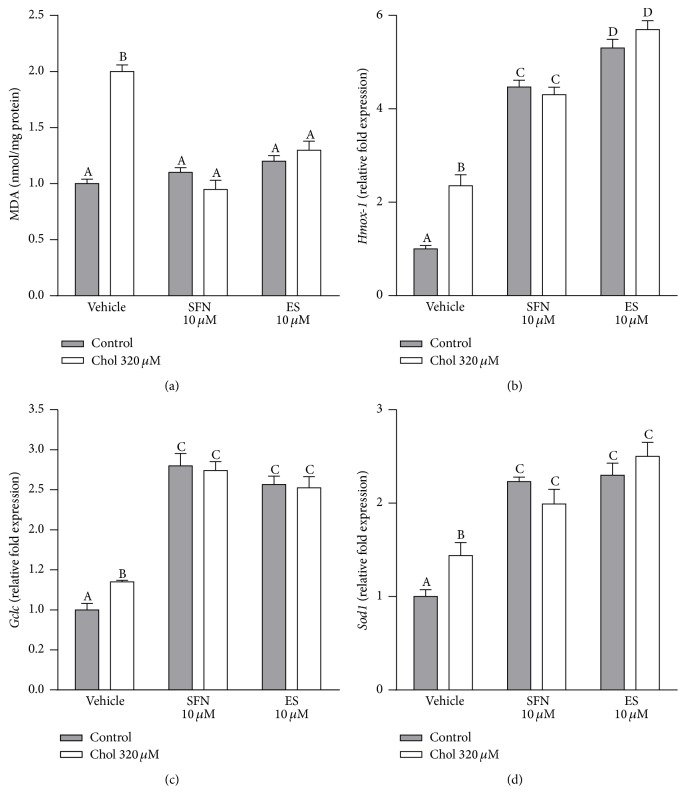
SFN prevents lipid peroxidation in Min6 cells exposed to cholesterol and induces the expression of genes related to antioxidant enzymes. (a) Oxidative stress was determined by measuring lipid oxidation using TBARS Assay Kit in Min6 cells incubated for 6 h with 320 *µ*M of cholesterol and/or 10 *µ*M of SFN or ES. Values are expressed as nmol of MDA/mg of protein. Gene expression of (b)* Hmox-1*, (c)* Gclc*, and (d)* Sod1* in Min6 cells treated for 20 h with of 320 *µ*M cholesterol and/or 10 *µ*M of SFN or ES was evaluated. Values are expressed as relative fold expression of two housekeeping genes (Table 2). Values are expressed as mean ± SEM, from three independent culture preparations, each treatment performed in quadruplicate. All two-ways ANOVAs and Bonferroni post-tests. Values with different superscript letters indicate significant differences (*p* < 0.05) between groups. Chol: cholesterol; SFN: sulforaphane; ES; 3,4-dihydroxyphenylacetic acid; MDA: malondialdehyde; Hmox-1: heme oxygenase-1; Gclc; glutamate-cysteine ligase catalytic subunit; and Sod1: Cu/Zn superoxide dismutase.

The exposure to cholesterol doubled lipid peroxidation in Min6 cells. This was completely averted in the presence of 10 *µ*M SFN or ES (two-way ANOVA, posttest *p* < 0.05, Figure 4(a)). SFN or ES at 10 *µ*M, in the absence of cholesterol, had no effect on lipid peroxidation. Cholesterol increased the gene expression of* Hmox-1*,* Gclc*, and* Sod1* by 135%, 35%, and 44%, respectively (all two-way ANOVAs, posttests *p* < 0.05, Figures 4(b)–4(d)). SFN or ES 10 *µ*M, with or without cholesterol, increased the expression of* Hmox-1 *by 4.4-fold and 5.5-fold, respectively (Figure 4(b)), the expression of* Gclc *by around 2.7-fold, and of* Sod1 *by around 2.2-fold (Figures 4(c) and 4(d)).

### 3.4. Sulforaphane Prevents Cholesterol-Induced Inflammation

**Figure 5:**
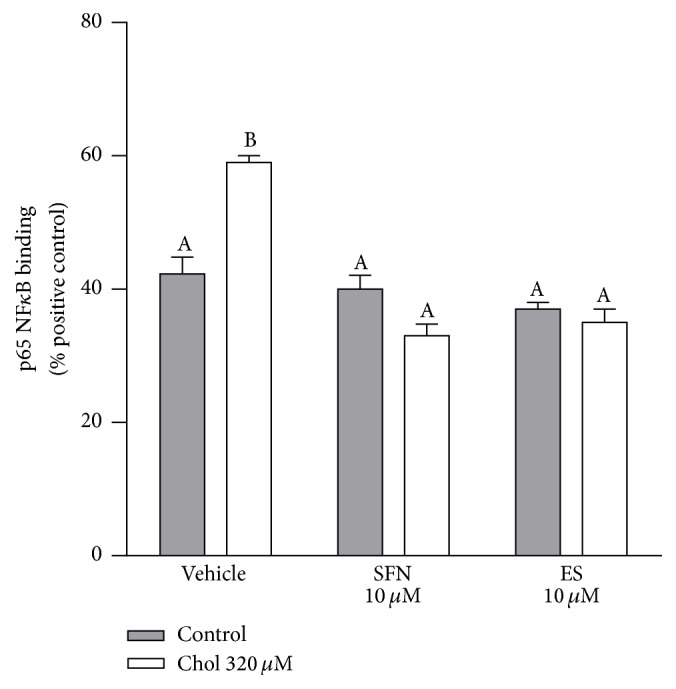
SFN inhibits the increase in nuclear NF*κ*B translocation in Min6 cells exposed to cholesterol. NF*κ*B (p65) binding to its cognate sequence was quantified in nuclear extracts from cells incubated for 6 h with 320 *µ*M of cholesterol and/or 10 *µ*M of SFN or ES. Values are expressed as percentage relative to positive control (HeLa cell lysate containing TNF*α*-activated NF*κ*B (p65), 100%). Values are expressed as mean ± SEM, from three independent culture preparations, each treatment performed in triplicate. All two-ways ANOVAs and Bonferroni post-tests. Values with different superscript letters indicate significant differences (*p* < 0.05) between groups. Chol: cholesterol; SFN: sulforaphane; and ES: 3,4-dihydroxyphenylacetic acid.

Cholesterol increased NF*κ*B translocation to the nucleus by 39%, being this effect prevented in the presence of 10 *µ*M SFN or ES (two-way ANOVA, posttest *p* < 0.05, Figure 5). In addition, cholesterol increased the levels of proinflammatory cytokines including IL-1*β*, TNF*α*, and IFN*γ* by 14%, 21%, and 17%, respectively, while it reduced the levels of anti-inflammatory cytokines such as IL-4 by 20% and IL-10 by 12% (All two-way ANOVAs, posttests *p* < 0.05, Table 1). In the presence of 10 *µ*M SFN or ES the cholesterol-induced alterations of cytokines levels were completely prevented (Table 1).

### 3.5. Sulforaphane Protects against Cholesterol Impaired Glucose-Stimulated Insulin Secretion

**Figure 6:**
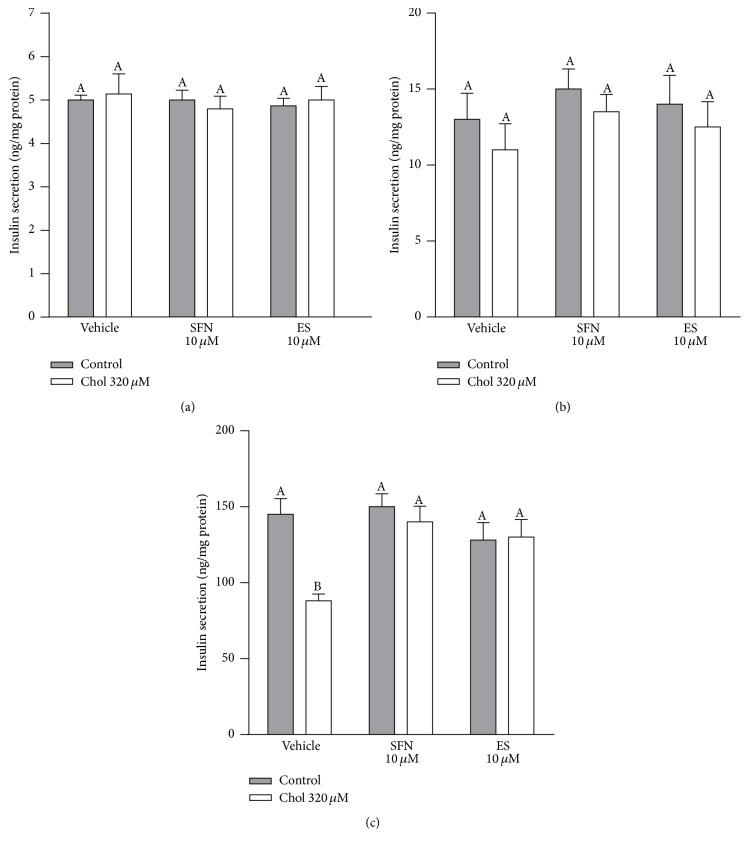
SFN protects against impaired glucose-stimulated insulin secretion in Min6 cells exposed to cholesterol. Insulin levels in the media of Min6 cells were determined in the (a) absence of glucose, (b) in response to 1 h exposure to low glucose (5 mM), or (c) high glucose (25 mM) after 6 h preincubation with 320 *µ*M of cholesterol and/or 10 *µ*M of SFN or ES, from three independent culture preparations, each treatment performed in triplicate. Values are expressed as mean ± SEM.* N* = 3. All two-ways ANOVAs and Bonferroni post-tests. Values with different superscript letters indicate significant differences (*p* < 0.05) between groups. Chol: cholesterol; SFN: sulforaphane; and ES: 3,4-dihydroxyphenylacetic acid.

Basal secretion of insulin (without glucose) was similar for all the treatments (4.97 ± 0.05 ng/mg protein, mean ± SEM, Figure 6(a)). Insulin secretion in control cells increased by 2.6-fold in response to low glucose compared to the secretion in the absence of glucose (Figures 6(a) and 6(b)). The insulin secretion in response to low glucose was not different between any of the treatments (13.2 ± 0.6 ng/mg protein, mean ± SEM, Figure 6(b)). Insulin secretion of control cells increased by 30-fold in response to high glucose, compared to the secretion in the absence of glucose (Figures 6(a) and 6(c)), and by 11-fold compared to the secretion stimulated by low glucose (Figures 6(b) and 6(c)). With high glucose, cholesterol caused a 40% decrease in insulin secretion compared to the same condition in control cells (two-way ANOVA, posttest *p* < 0.05, Figure 6(c)). This effect of cholesterol was completely prevented by 10 *µ*M SFN or ES (Figure 6(c)). SFN or ES alone had no effect on the insulin secretion induced by high glucose (Figure 6(c)).

## 4. Discussion

Pancreatic *β*-cell dysfunction, leading to an impaired insulin secretory response to glucose, plays a pivotal role in the transition from prediabetic state to the clinical type 2 diabetes mellitus (T2DM) [9, 26, 27]. In this study we found that SFN, to the same extent as ES, prevented GSIS impairment in a pancreatic *β*-cell line exposed to cholesterol. We demonstrated that SFN prevented cholesterol-induced mitochondrial bioenergetic dysfunctions, oxidative stress, and inflammation. The novel mechanisms of bioenergetics regulation by SFN described in the present study suggest that SFN is a potent protective agent of pancreatic *β*-cell function.

### 4.1. Sulforaphane Protects against Mitochondrial Dysfunctions Induced by Cholesterol

Our results demonstrate that cholesterol induces mitochondrial dysfunction by interfering with the ETC (Figures 2(a)–2(d)). We have recently shown that cholesterol reduces the activity of complex I [7], and in this study, we also showed for the first time that cholesterol impairs the respiration driven by complexes I, II, and IV during a state of major energy requirement induced by an uncoupler (further discussed below). A slowed electron flow reduces oxygen consumption and oxidative phosphorylation in mitochondria [28] as shown by the decrease in basal and maximal respiration, spare capacity, ATP turnover, and coupling efficiency observed in Min6 cells treated with cholesterol (Figures 1(c)–1(e)). Sulforaphane not only prevented this mitochondrial dysfunction, but also improved basal and maximal respiration as well as spare capacity and ATP turnover in Min6 cells. The protective effects of SFN on mitochondrial respiration may rely on its ability to improve complexes I-, II-, and IV-driven respiration. Although SFN has been previously shown to display hepatoprotective effects by preserving mitochondrial function, specifically the activities of mitochondrial complexes [29], here we have demonstrated for the first time enhanced respiration driven through complexes I, II, and IV by SFN in the pancreatic *β*-cell line and during increased energy demand. The latter is highly relevant in insulinoma, specifically under hyperglycemia condition, as *β*-cells constantly undergo an increased energy demand when insulin needs to be released. In addition, the spare capacity appears to be an important diagnostic measure of cell bioenergetics that experience high fluctuation in ATP demand [30]. SFN improves bioenergetics of the cells during high energy demands, with an increased spare capacity and an efficient electron flow resulting in higher ATP turnover. An elevation in the ATP/ADP ratio ensures continued exocytosis of insulin [31]. This is one important mechanism for the protection of SFN against the impairment on GSIS induced by cholesterol (Figure 6(c)).

### 4.2. Sulforaphane Improves the Expression of Genes Related to Mitochondrial Function

Our results also show that the improvement in mitochondrial function induced by SFN was associated with a rise in* Sirt1* (Figure 3(a)) and* Pgc-1α* expression (Figure 3(b)) in both vehicle and cholesterol treated cells. Sulforaphane was shown to prevent the decreased expression of* Sirt1* and* Pgc-1α* in an animal model of T2DM, as a protective mechanism against diabetic cardiomyopathy [32] and muscle atrophy [33]. However, this is the first study evaluating the modulation of the expression of these genes by SFN as another mechanism for the protection of pancreatic *β*-cells. In *β*-cells, Sirt1 regulates the expression of specific mitochondria-related genes that control metabolic coupling and a decrease in* Sirt1* expression impairs glucose sensing and insulin secretion [34]. Sirtuin 1 activates PGC-1*α* by deacetylation, a key regulator of mitochondrial biogenesis and function [35]. Sulforaphane also promoted* Pgc-1α* expression (Figure 3(b)), probably through the increased expression of* Sirt1* as evidenced by the improved mitochondrial function observed in the presence of SFN (Figures 1(a)–1(h) and Figures 2(a)–2(d)). In contrast, the increase in* Pgc-1α* expression induced by cholesterol (Figure 3(b)) may reflect a compensatory mechanism due to decreased levels of* Sirt1* and thus lower activation of PGC-1*α*, which is reflected by the decreased mitochondrial activity (Figures 1(a)–1(h) and Figures 2(a)–2(d)). Alternatively, it might be an effort to directly restore the diminished energetic levels of the cell.

### 4.3. Sulforaphane Protects against the Oxidative Stress Induced by Cholesterol

Interestingly, we found that cholesterol, and to a higher extent SFN, induced the expression of antioxidant genes including* Hmox-1, Gclc, *and* Sod1* and downstream Nrf2 activation (Figures 4(b)–4(d)). However cholesterol increased lipid peroxidation while SFN protected against this deleterious effect (Figure 4(a)). We have previously shown that cholesterol increases ROS levels and reduces SOD and glutathione peroxidase activities in Min6 cells, despite of increasing the translocation of Nrf2 to the nucleus [7]. This suggests that cholesterol promotes the inactivation of the antioxidant defences downstream Nrf2 activation by promoting the oxidation of the catalytic moieties of the antioxidant enzymes [36–38]. This is supported by the fact that, in the presence of an antioxidant such as ES, the cholesterol-induced oxidative stress as well as the inactivation of the antioxidant enzymes was totally prevented [7].

SFN, however, restored the redox homeostasis within the cell by increasing the expression of the antioxidant enzymes downstream Nrf2 activation (Figures 4(b)–4(d)) and, unlike cholesterol, preserved their activities by blocking oxidative stress, an event which is consistent with the protective effect of SFN on lipid peroxidation induced by cholesterol (Figure 4(a)). Interestingly, cobalt protoporphyrin, a known* Hmox-1* inducer, protected INS-1 *β*-cells from high glucose-induced oxidative stress and apoptosis; however, it failed in restoring the GSIS in these cells [39]. The latter indicates that the mechanisms underlying the protective effect of SFN against the pancreatic *β*-cells dysfunction induced by cholesterol are beyond Nrf2 pathway induction and oxidative stress inhibition. The protective effects on mitochondrial bioenergetics provide a plausible mechanism supporting the antioxidant effects of SFN.

### 4.4. Sulforaphane Protects against Inflammation Induced by Cholesterol

SFN prevented the cholesterol-induced nuclear translocation of NF*κ*B (Figure 5) and the increase in proinflammatory cytokine and the decrease in anti-inflammatory cytokine levels (Table 1). Therefore, SFN may protect against mitochondrial and pancreatic *β*-cells dysfunction by preventing inflammation. A combination of inflammatory cytokines (IL-1*β*, IFN*γ*, and TNF-*α*) decreases GSIS and promotes oxidative stress and mitochondrial dysfunction in INS-1 and RINm5F *β*-cells [40, 41]. SFN may protect *β*-cells via Hmox-1 (Figure 4(b)), since a cell-permeable heme oxygenase-1 (PEP-Hmox-1) protected INS-1 *β*-cells against apoptosis, oxidative stress, and inflammation induced by a cytokine mixture (IL-1*β*, IFN*γ*, and TNF-*α*) [42]. The antioxidant properties of SFN might also contribute to the prevention of cholesterol-induced activation of the NF*κ*B pathway observed in this study, since ROS are associated with NF*κ*B activation through the increase in I*κ*B degradation [43]. SFN may also protect *β*-cells via Sirt1 (Figure 3(a)), since Sirt1 inhibits the transcriptional activity of NF*κ*B, by deacetylating the RelA/p65 subunit [44] and* Sirt1* overexpression downregulates NF*κ*B activity in mice [45] and improves GSIS in *β*-cells [46].

### 4.5. Sulforaphane Protects Pancreatic Beta Cells against Cholesterol to the Same Extent as 3,4-Dihydroxyphenylacetic Acid

3,4-dihydroxyphenylacetic acid (ES) is the major microbial metabolite of quercetin and its glycosylated derivatives [47–54]. The flavonol quercetin is one of the most abundant polyphenol present in fruit and vegetables and in the Western diet [55–57]. It has been shown that ES is absorbable [58–60] and exerts strong antioxidant properties directly by scavenging free radicals [61] or indirectly by inducing the Nrf2 pathway [7, 62]. ES has been shown to protect against pancreatic *β*-cells dysfunction induced by cholesterol through its antioxidant and mitochondrial protective properties [7]. Our results show that ES and SFN exhibit similar effects, improving mitochondrial respiration during basal and high energetic demands by improving the electron flow through the ETC (Figures 1(a)–1(h) and Figures 2(a)–2(d)). Their anti-inflammatory activities (Figure 5 and Table 1) also appear to contribute to the protection against pancreatic *β*-cells dysfunction induced by cholesterol.

### 4.6. In Vivo Approach of the Health Effects of Sulforaphane and 3,4-Dihydroxyphenylacetic Acid in Terms of Their Effective Concentrations

Studies in both humans and rodents [63–65] have demonstrated that the intake of cruciferous vegetable containing active myrosinase results in a higher production of isothiocyanates than that of cruciferous lacking active myrosinase. For example, a plasma SFN concentration of 4 nM was detected in human volunteers after the intake of 200 g of blanched broccoli for 4 weeks [66] while a substantially higher plasma concentration of 2.3 *µ*M of SFN was observed after consuming 40 g of fresh broccoli sprouts [67]. This can be explained by the inactivation of myrosinase during cooking. Increased SFN bioavailability can be attained after the intake of SFN-enriched broccoli sprout preparation (generated by quick steaming followed by myrosinase treatment) in mice [68]. Although the concentration of SFN used in our study cannot be reached after consuming broccoli in a normal diet, it could be attained through the intake of a diet rich in myrosinase-treated broccoli or SFN-based nutraceuticals.

It is unlikely that the effective concentration of ES studied in vitro here can be reached in vivo. However, additional experiments are needed to establish the plasma concentration that could be reached with a standard diet or a polyphenol rich diet, since the available information is mainly based on studies with limited sample sizes or carried out with subjects eating uncontrolled diets [58–60, 69, 70]. It seems that most of the ES absorbed is metabolized to 3-methoxy-4-hydroxyphenylacetic acid, since the concentration of this methyl derivative was found to be 24-fold higher than ES in the 24 h urine collections [60]. Thus, the intermediate metabolism is another important variable to consider since flavonoid bioavailability is intrinsically regulated by factors such as dietary intake, differences in host microbiota, polymorphism of intestinal transporters, metabolic pathways, and excretion. Moreover, the metabolic activity of the microbiota from diabetic subjects remains to be studied, specifically in terms of polyphenol metabolism.

In conclusion, our study demonstrates that SFN protects *β*-cells against cholesterol-induced impairments of their mitochondrial function by improving the electron flow in the ETC as well as the basal and maximal respiration, spare capacity, and ATP turnover. SFN promotes the expression of genes involved in antioxidant defense and averts cholesterol-induced lipid peroxidation and activation of the NF*κ*B pathway, normalizing the expression of pro- and anti-inflammatory cytokines. The deleterious effects of cholesterol are associated with a decrease in* Sirt1 *expression while SFN increases it. These actions of SFN are similar to those of ES and are well suited to protect against cholesterol-induced pancreatic *β*-cell dysfunction, thereby preserving GSIS. Our results indicate that sulforaphane and ES are protective agents against cholesterol-induced alterations of pancreatic *β*-cell function. This study supports the consumption of glucosinolates and polyphenol-containing nutraceuticals, fruit, and vegetables to reduce the risk of diabetes.

## Figures and Tables

**Table 1:** Sulforaphane and ES prevent the changes in cytokines levels induced by cholesterol in Min6 cells.

	pg cytokines/mg protein				
	IL-1*β*	TNF*α*	IFN*γ*	IL-4	IL-10
Control	74.15 ± 0.43^A^	15.03 ± 0.40^A^	31.22 ± 0.57^A^	14.33 ± 0.59^A^	85.20 ± 1.49^A^
Chol 320 *µ*M	82.02 ± 0.55^B^	18.23 ± 1.85^B^	36.66 ± 1.24^B^	11.42 ± 1.10^B^	75.13 ± 1.49^B^
SFN 10 *µ*M	69.90 ± 0.87^A^	12.85 ± 0.25^C^	27.51 ± 0.26^A^	18.40 ± 0.58^C^	89.00 ± 1.31^A^
SFN 10 *µ*M + chol 320 *µ*M	65.25 ± 1.30^C^	12.57 ± 0.35^C^	29.23 ± 1.20^A^	20.00 ± 0.22^C^	85.32 ± 1.11^A^
ES 10 *µ*M	71.78 ± 1.16^A^	11.86 ± 0.44^C^	28.56 ± 0.34^A^	16.39 ± 0.68^D^	90.60 ± 1.64^A^
ES 10 *µ*M + chol 320 *µ*M	71.28 ± 1.92^A^	12.56 ± 0.38^C^	28.50 ± 1.66^A^	17.89 ± 0.39^D^	86.25 ± 1.34^A^

Proinflammatory and anti-inflammatory cytokines in Min6 cells treated for 20 h with 320 *µ*M cholesterol and/or 10 *µ*M SFN or ES. Values are expressed as mean ± SEM, from three independent culture preparations, each treatment performed in quadruplicate. All two-way ANOVA and Bonferroni posttests. Values with different superscript letters indicate significant differences (*p* < 0.05) between groups.

**Table 2:** List of primers used in this study for RT-PCR.

Gene	Forward	Reverse
*Sirt1*	GTTGATTGTGAAGCTGTTCGTG	TGGCTCTATGAAACTGTTCTGG
*Pgc-1α*	CACCAAACCCACAGAAAACAG	GGGTCAGAGGAAGAGATAAAGTTG
*Hmox-1*	GTTCAAACAGCTCTATCGTGC	TCTTTGTGTTCCTCTGTCAGC
*Gclc*	CTCCAGTTCCTGCACATCTAC	AGAACATCGCCTCCATTCAG
*Sod1*	AAGACTGGAAATGCTGGGAG	GGTTTGAGGGTAGCAGATGAG
*Tbp*	TTCTCGAAAGAATTGCGCTGT	GCCTTGTGAGTCATTTCAGTGA
*Hmbs*	AAGGGCTTTTCTGAGGCACC	AGTTGCCCATCTTTCATCACTG

Hmbs: hydroxymethylbilane synthase; Hmox-1: heme oxygenase-1; Gclc: glutamate-cysteine ligase catalytic subunit; Pgc-1*α*: peroxisome proliferator-activated receptor gamma coactivator-1-alpha; Sirt1: sirtuin 1; Sod1: Cu/Zn superoxide dismutase; and Tbp: TATA box binding protein.
